# Report of two siblings with APECED in Serbia: is there a founder effect of c.769C>T *AIRE* genotype?

**DOI:** 10.1186/s13052-021-01075-8

**Published:** 2021-06-02

**Authors:** Alessandra Fierabracci, Mariafrancesca Lanzillotta, Ivana Vorgučin, Alessia Palma, Dragan Katanić, Corrado Betterle

**Affiliations:** 1 Infectivology and Clinical Trials Research Department, Bambino Gesù Children’s Hospital, IRCCS, Rome, Italy; 2 Institute for Child and Youth Health Care of Vojvodina, Faculty of Medicine Novi Sad, Vojvodina, Serbia; 3 Research Laboratories, Bambino Gesù Children’s Hospital, IRCCS, Rome, Italy; 4 Endocrine Unit, Department of Medicine (DIMED), University of Padua, Padua, Italy

**Keywords:** Autoimmune polyglandular syndrome type 1, APECED, *AIRE*, Serbian population, Genotype-phenotype variability, Autoantibodies

## Abstract

**Background:**

Autoimmune polyendocrinopathy-candidiasis-ectodermal-dystrophy (APECED) or autoimmune polyglandular syndrome Type 1 is a rare autosomal recessive syndrome. The disorder is caused by mutations in the *AIRE* (AutoImmune Regulator) gene. According to the classic criteria, clinical diagnosis requires the presence of at least two of three main components: chronic mucocutaneous candidiasis, hypoparathyroidism and primary adrenal insufficiency. Furthermore, patients are often affected by other endocrine or non-endocrine associated autoimmune conditions. The enrichment of the non-classical triad seems to occur differently in different cohorts. Screenings of the population revealed that homozygous *AIRE* mutations c.769C > T, c.415C > T and c.254A > G have a founder effect in Finnish, Sardinian and Iranian Jew populations respectively.

**Case presentation:**

We report here the clinical and genetic characteristics of two new Serbian APECED siblings, one male and one female, actual age of 27 and 24 respectively, born from non-consanguineous parents. Addison’s disease was diagnosed in the male at the age of 3.5 and hypoparathyroidism at the age of 4. The female developed hypoparathyroidism at 4 years of age. She presented diffuse alopecia, madarosis, onychomycosis, teeth enamel dysplasia. She further developed Addison’s disease at the age of 11 and Hashimoto’s thyroiditis at the age of 13.5. She had menarche at the age of 14 but developed autoimmune oophoritis and premature ovarian failure at the age of 16. A treatment with hydrocortisone, fludrocortisone and alfacalcidiol was established for both siblings; L-T4 (levo-thyroxine) for thyroid dysfunction and levonorgestrel and etinilestradiol for POF were also administered to the female.

Genetic screening revealed a homozygous c.769C > T (R257X (p.Arg257X)) *AIRE* mutation. We additionally reviewed the literature on 11 previously published Serbian patients and evaluated the frequency of their main diseases in comparison to Finnish, Sardinian, Turkish, Indian and North/South American cohorts.

**Conclusion:**

A founder effect was discovered for the R257X genotype detected in the DNA of 10 homozygous and 2 heterozygous patients. Of note, all Serbian APECED patients were affected by adrenal insufficiency and 10 out of 13 patients presented CMC.

## Introduction

Autoimmune polyendocrinopathy-candidiasis-ectodermal-dystrophy (APECED) also known as autoimmune polyglandular syndrome Type 1 (APS-1) [1] is a rare autosomal recessive disorder caused by mutations in the Autoimmune Regulator (*AIRE*) gene [2].

According to the classic criteria, clinical diagnosis of APECED is based on the presence of at least two of 3 main disorders, i.e. chronic mucocutaneous candidiasis (CMC), chronic hypoparathyroidism (CH) and primary adrenal insufficiency (autoimmune Addison’s disease, AAD). During their lifetime, patients may also develop other autoimmune endocrine conditions such as thyroiditis, Type 1 diabetes, hypophysitis, hypergonadotropic hypogonadism, and non-endocrine autoimmune diseases such as gastritis, celiac disease, intestinal disorders and ectodermal dystrophy [3]. In America, APECED patients present a dramatic development of non-classical triad manifestations in early life in comparison to European cohorts [4].

Epidemiological investigations from different countries demonstrate that the overall prevalence of the disease is lower than 10/million population. Genetically restricted ethnic groups including Iranian Jews (1/9000), Sardinians (1/14,000), Finns (1/25,000) and Slovenians (1/43,000) (reviewed (rev) in [3]) show a higher prevalence of disease whilst Norwegians (1/80,000), Irish (1/130,000) and Polish (1/129,000) present a lower prevalence (rev in [3, 5]). Furthermore, APECED is rare in the French (1/500,000) and Japanese (1/10 million) populations (rev in [3, 6–9]). It is estimated that in Italy there are approximately 200 APECED patients with an overall prevalence of 3–3.5/million population [3]. In particular, within the Italian territory there are 3 hot spots with a high incidence of APECED: Sardinia (Ogliastra) (1/14,000), Apulia (Salento) (1/35,000) and Veneto (Bassano del Grappa) (1/4400) [10, 11].

Homozygous *AIRE* mutations c.769C > T, c.415C > T and c.254A > G have a founder effect in the Finnish, Sardinian and Iranian Jew populations respectively [12]. Furthermore, patients from the same population may harbor the same APECED-causing mutation which however correlates with a high phenotypic variability. Two autosomal dominant mutations of *AIRE* have also been reported [13, 14]. Indeed, the G228W mutation was discovered in a patient from Tuscany affected by CH and autoimmune thyroiditis with the presence of circulating 21OH antibodies [13]; whilst the c.932G > A (p.C311Y) *AIRE* variant was found in a North-African patient affected by CMC, AAD, enamel dystrophy, partial diabetes insipidus and pernicious anemia [14].

Here we report the clinical and genetic characteristics of two new Serbian APECED patients and reviewed the literature on other Serbian APECED patients.

## Case presentation

Two siblings, one male and one female (actual age of 27 and 24 years (yr) respectively), born from non-consanguineous parents, are reported; they have respectively developed 2 and 7 diseases. Addison’s disease was diagnosed in the male at the age of 3.5 and hypoparathyroidism at the age of 4. His thyroid function tested normal at the age of 19. The female developed hypoparathyroidism at the age of 4. She presented diffuse alopecia, madarosis, onychomycosis, teeth enamel dysplasia. Addison’s disease was diagnosed at the age of 11 and Hashimoto’s thyroiditis at the age of 13.5. She had menarche at the age of 14 but developed autoimmune oophoritis and premature ovarian failure (POF) at the age of 16. Anti-thyroid peroxidase (TPO) and anti-ovarian antibodies tested positive. Hydrocortisone, fludrocortisone and alfacalcidiol treatment was established in both siblings; L-T4 (levo-thyroxine) for thyroid dysfunction and levonorgestrel and etinilestradiol for POF were also administered to the female.

Genetic screening revealed a homozygous c.769C > T (R257X (p.Arg257X)) mutation. The parents of the children gave informed consent to publish their anonymized details at the Institute for Child and Youth Health Care of Vojvodina, Faculty of Medicine Novi Sad, Vojvodina-Serbia (Table 1 cases 1. 2).

**Table 1:** Clinical and genetic characteristics of the series of 13 Serbian APECED patients including two novel case reports

Patient	Sex/age at referral (years)	Age of first symptom (years)	***AIRE*** mutation	Major clinical manifestations related to APECED^**e**^	Other clinical manifestations^**e**^	Reference
^a, d^1.	M/3.5	3.5	c.769C > T R257X (p.Arg257X)	Addison’s disease (3.5) Hypoparathyroidism (4)		Present report
^a, d^2.	F/4	4	c.769C > T R257X (p.Arg257X)	Mucocutaneous candidiasis Hypoparathyroidism (4) Addison’s disease (11)	Ectodermal dystrophy Diffuse alopecia with madarosis Onychomycosis Hashimoto’s thyroiditis (13.5) POF (16)	Present report
^a, c, d^3.	F/33	7	c.769C > T R257X (p.Arg257X)	Hypoparathyroidism (7) Addison’s disease (14) Mucocutaneous candidiasis (16)	Pure red cell aplasia Hashimoto’s thyroiditis POF (30) Renal dysfunction Exocrine pancreas insufficiency (36)	[15]
4.(younger sister of patient 3)	F		c.769C > T R257X (p.Arg257X)	Chronic mucocutaneous candidiasis Hypoparathyroidism Addison’s disease	Pernicious anemia *Lichen ruber planus*	[15]
^a, d^5.			c.769C > T R257X (p.Arg257X)	Mucocutaneous candidiasis Addison’s disease	Vitiligo Alopecia Ectodermal dystrophy Autoimmune hepatitis	[16]
6.	F/21	7.5	p.Glu298Lys/ p.Arg257X c.892G > A/ c.769C > T	Hypoparathyroidism (7.5) Addison’s disease (8) Mucocutaneous candidiasis (11)	Autoimmune bronchiolitis (3.5) Hypogonadism (12) Chronic Otitis media with effusion (16.5) Systemic Juvenile Rheumatoid Arthritis Pernicious anemia (17)	[17]
^a, b, c^7.	M/12	2	p. (=)/ p.Arg257X c.462A > T/ c.769C > T	Addison’s disease (11)	Ectodermal dystrophy (2) Malabsorption (2) Vitiligo (2) Alopecia (2.5)	[17]
8.	F/8	1	c.769C > T R257X (p.Arg257X)	Mucocutaneous candidiasis (1) Addison’s disease (4) Hypoparathyroidism (5)	Vitiligo (1)	[17]
^a, c, d^9.	F/22	5	c.769C > T R257X (p.Arg257X)	Mucocutaneous candidiasis (5) Addison’s disease (16) Hypoparathyroidism (15)	Chronic hepatitis (15) Pernicious anemia (16)	[17]
^a, b, c^10.	M/20	6	c.769C > T R257X (p. Arg257X)	Mucocutaneous candidiasis (6) Hypoparathyroidism (9) Addison’s disease (11)	Malabsorption (9)	[17]
^a, c^11.	F/23	9	c.769C > T R257X (p.Arg257X)	Hypoparathyroidism (9) Mucocutaneous candidiasis (10) Addison’s disease (10)	Alopecia (10) Hypogonadism (18)	[17]
^a, b, c^12.	M/19	10	c.769C > T R257X (p.Arg257X)	Mucocutaneous candidiasis (11) Hypoparathyroidism (13) Addison’s disease (14)	Pernicious anemia (10) Malabsorption (11) Alopecia (13)	[17]
^a, c^13.	F/20	15	not available	Adrenal insufficiency (15) Hypoparathyroidism (15)	Graves’ disease (15) Vitiligo (15) POF (15) Alopecia *universalis* (18) Vogt-Koyanagi-Harada syndrome (20)	[18]

^a^APECED patients for which sufficient information is retrospectively available to support a clinical diagnosis also based on the presence of Ferre/Lionakis criteria (i.e., presence of one symptom of the classic triad and one symptom of the adjunct triad of urticarial eruption, intestinal dysfunction and enamel hypoplasia). *N* = 10

^b^Patients for which sufficient information is retrospectively available and it was possible to confirm an earlier diagnosis based on Ferre/Lionakis criteria versus classic criteria. *N* = 3

^c^Patients for which sufficient information is retrospectively available to verify whether an earlier diagnosis based on Ferre/Lionakis criteria versus classic criteria was possible. *N* = 7

^d^Incompletely evaluable patients for an earlier diagnosis based on Ferre/Lionakis criteria versus classic criteria due to lack of information on the age of appearance of symptoms. *N* = 5

^e^Age of appearance of symptom (years)

## Discussion

### Analysis of clinical and genetic features in the Serbian APECED population

We discuss the features of the two new siblings by retrospectively evaluating the clinical and genetic characteristics of other 11 Serbian APECED patients (10 of which with available confirmatory *AIRE* genotype) reported in literature from 2001 till date (Table 1). The data are compared with those retrieved from North (Finland) [19] and South of Europe (Sardinia) [20], North and South America [4], Turkey [21] and India [22].

### Clinical manifestations

**Fig. 1:**
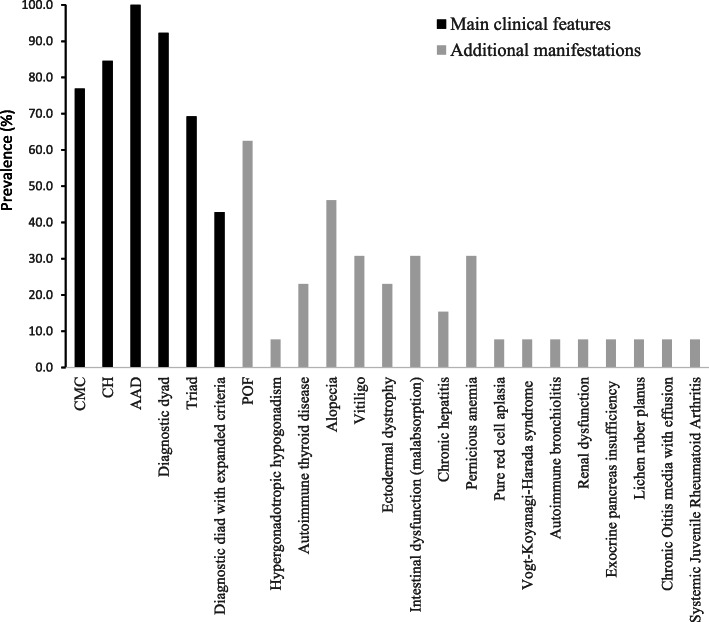
The prevalence (%) of the various clinical manifestations seen in the Serbian cohort (*n* = 13)

**Fig. 2:**
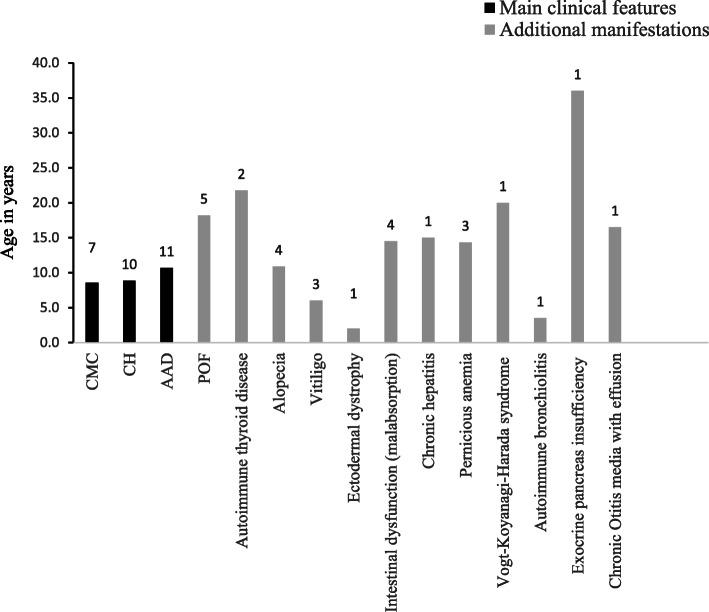
The mean age of appearance of main and secondary clinical manifestations in the present Serbian APECED series. On top of each bar is reported the number of APECED subjects for each clinical disease manifestation (among those whose age of onset is known)

The details of the total cohort of 13 Serbian APECED patients, clinically diagnosed based on the classic criteria, are given in Table 1 [15–18]. The female/male ratio was 8/4, median age at referral was 16.9 yr (range 3.5–33 yr) and early disease onset had a median age of 6.36 yr (range 1–15 yr), based on the appearance of the first component of the triad and the severity of the phenotype. At the end of the observation period, the classical triad was present in 9 of the 13 patients and the dyad in 12 of the 13 patients. A mean of 5.53 manifestations were reported per patient and the frequencies of the diseases are summarized in Fig. 1. Figure 2 refers to the mean age of appearance of clinical manifestations.

Epidemiological investigations conducted on different populations demonstrate that CMC is usually the first manifestation of APECED, often before 5 years of age [19, 23]. The frequency of CMC ranges between 17 and 100% in APECED patients in different series, with the lowest incidence in Iranian Jews [23]. CH is the second most common manifestation in order of appearance (rev in [3, 8, 11, 19, 23]) whilst AAD is usually the third manifestation, occurring in 22–95% of the patients (rev in [8, 11]). Concurrently, APECED patients suffer additional endocrine and non-endocrine autoimmune conditions [2, 3].

**Fig. 3:**
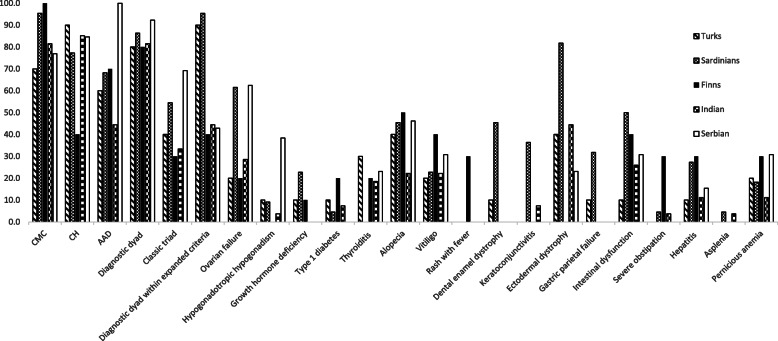
Disease components of the Serbian series, considering patients with genetically confirmed diagnosis, compared to other populations. Prevalence of diagnostic dyad/triad and the most common APECED disease components in Serbian patients (*n* = 13), Indian patients (*n* = 27), Turkish (*n* = 10), Sardinian (*n* = 22) and the Finnish series (*n* = 10)

Among the 13 Serbian patients described, in 7/13 whose age of onset was known (53.8%) CMC was the first disease to manifest, isolated or in association with other symptoms, with a mean age of onset of 8.57 yr (range 1–16 yr) (Table 1). Of the 13, 10 (76.9%) exhibited CMC (2 males, 7 females based on available data) at some stage after the onset of the disease (Fig. 1). Among patients diagnosed before 30 yr of age and genetically confirmed by *AIRE* gene screening, CMC was present in 10/13 (76.9%) of Serbian patients compared to 81.48% of Indian patients [22], 70% Turkish [21], 95.5% Sardinian [20] and all Finnish patients [19] (Fig. 3).

Of the 13 patients, 84.6% (11/13) developed CH (3 males, 8 females) (Fig.1). CH, as the first disease to manifest, isolated or in association with other symptoms, had a mean age of onset of 8.85 yr (range 4–15 yr). According to published data, CH develops in 50–100% of APECED patients worldwide [8, 11]. Among patients diagnosed before the age of 30 (12/13 with clinical diagnosis confirmed by *AIRE* gene screening), CH was found in 11/13 (84.6%) of the Serbian patients, 23/27 (85.1%) Indian patients [22], 90% Turks [21] and 77.3% Sardinians [20] but only in 40% of Finnish patients [19] (Fig. 3).

In the Serbian APECED series, 100% of the patients were affected by AAD; this occurred as first manifestation (isolated or with other symptoms) in 11/13 (84.6%) patients at a mean age of 10.6 for those whose age of disease onset was available (range 3.5–16 yr). On the whole, 100% (13/13) of Serbian, 44.44% Indian [22], 60% Turkish [21], 68.2% Sardinian [20] and 70% Finnish [19] patients with genetic diagnosis suffered from AAD (Fig. 3).

Other endocrinopathies include POF that manifests as primary or secondary amenorrhea with hypergonadotropic hypogonadism in 0–71% of cases according to national surveys (rev in [8, 11]). Among Serbians, OF was present in 5/8 of the evaluated females (62.5%), 28.5% Indians [22], 20% Turks [21], but nearly two thirds (61.5%) of Sardinians [20] (Fig. 3). Autoimmune thyroid disease (AITD), mostly Hashimoto’s thyroiditis and rarely Graves’ disease, was diagnosed in 29% of all reported APECED patients at a median age of 19 yr (rev in [8, 11]). Among Serbians AITD was reported in 3/13 (23%) (Hashimoto’s thyroiditis in 2 patients and Graves’ disease in one patient), 18.5% (5/27) Indians [22], 30% Turks [21], 20% Finns [19] but none of the Sardinians [20] (Fig. 3).

A higher incidence (40–80%) of non-endocrine manifestations was reported in North American APECED patients compared to < 5–20% in some other cohorts [4]. Non-endocrine manifestations encountered in the Ferre/Lionakis criteria help accelerate early diagnosis of APECED in the American cohort [4] compared to the classic criteria. These expanded criteria for clinical diagnosis are fulfilled if patients develop 2 out of 6 manifestations, which include non-endocrine urticarial eruption, intestinal dysfunction and enamel hypoplasia, along with the classic components CMC, AAD and CH [4]. In the Serbian series, a high incidence of non-endocrine manifestations was reported in 12 out of 13 patients with a mean of 2 manifestations per patient. These included ectodermal dystrophy (*n* = 3, patient 2, 5 and 7, Table 1), alopecia (*n* = 6, patient 5, 7, 11 and 12, diffuse alopecia with madarosis in patient 2, alopecia *universalis *in patient 13), pure red cell aplasia (patient 3), renal dysfunction (patient 3), exocrine pancreas insufficiency (patient 3), pernicious anemia (*n* = 4, patient 4, 6, 9, 12), *Lichen ruber planus* (patient 4), chronic otitis media with effusion (patient 6), autoimmune bronchiolitis manifested as asthma-like dyspnea (patient 6), Systemic Juvenile Rheumatoid Arthritis (patient 6), vitiligo (*n* = 4, patient 5, 7, 8 and 13), chronic active hepatitis (patient 5 and 9) and malabsorption (*n* = 4, patient 3, 7, 10 and 12) [15–17]. Based on the limited retrospective information available, the expanded criteria [4] allowed an early diagnosis in only 3 patients (patients 7, 10, 12 in Table 1) out of 9 whose information was sufficient for evaluation. Prospective studies conducted on a larger number of patients will be helpful to compare the diagnostic efficacy of these two sets of criteria.

Among the non-endocrine manifestations, ectodermal dystrophy was present in 3 out of 13 (23%) patients from the Serbian cohort (1 male, 1 female based on the available information) (Fig. 1), 12/27 (44.4%) Indian [22], 4/10 (40%) Turkish [21], 81.8% Sardinian [20] and absent in Finnish patients [19] (Fig. 3). Alopecia was present in 6/13 Serbian patients (46%), progressing to alopecia *universalis* in one female. Vitiligo has been reported in 6–50% and alopecia *areata* in 7–52% APECED patients in different studies [8, 11]. Vitiligo was observed in 4/13 (30%) Serbian patients (one male, 2 females based on the available information) (Fig. 1), 6/27 (22.2%) Indian [22], 2/10 (20%) Turkish [21], 22.7% Sardinian [20] and 40% Finnish patients [19] (Fig. 3).

Autoimmune gastritis, manifesting as mucosal atrophy with submucosal lymphocytic infiltration and loss of parietal cells, is reported in 4–32% APECED patients worldwide, either in isolation or in association with pernicious anemia (rev in [23]). Gastric parietal cell failure was not documented in the Serbian series (Table 1), though pernicious anemia affected 4 out of the 13 (30%) (patient 4, 6, 9, 12 in Table 1) APECED patients (Fig. 1). For comparison, pernicious anemia was reported in 11.1% Indian, 20% Turkish, 18.2% Sardinians, and 30% Finnish patients [19–22] (Fig. 3).

Intestinal dysfunction in APECED can be due to rare conditions like celiac disease, exocrine pancreas insufficiency, intestinal lymphangiectasia, or intestinal infections such as *Candida albicans, Giardia lamblia, Clostridium difficile* etc. There is also evidence of an autoimmune origin associated with the presence of circulating antibodies against tryptophan hydroxylase (TPHAbs), histidine hydroxylase (HD) or L-amino acid decarboxylase (AADC) Abs [1, 24, 25]. Its incidence varies between 15 and 22% in different series (rev in [23]). Intestinal dysfunction appeared in 4/13 (30.7%) Serbian patients, 25.9% Indian [22], 10% Turkish [21], 50% Sardinian [20] and 40% Finnish [19] patients (Fig. 3).

Additional rare manifestations reported in previous studies (rev in [23]) were present in a few Serbian APECED patients. Among these, pure red cell aplasia manifested in patient 3 (see above); it is a rare syndrome characterized by severe normocytic anemia, reticulocytopenia, and the absence of detectable erythroid precursors in the bone marrow [26]. Few APECED patients have been reported in literature with this associated autoimmune disorder due to the presence of autoantibodies against erythroid cells or erythropoietin [15, 26]. Hyperactivity of T cells, especially of large granular subsets, or NK cells was also considered a possible pathogenic mechanism [15]. Another rare condition represented in the Serbian series is the Vogt-Koyanagi-Harada syndrome manifesting with uveitis, right hypoacusia and right hemiparesis (patient 13) (Table 1) [18].

### Genotype/phenotype correlation

APECED is a monogenic recessive syndrome with 100% penetrance. The inheritance of two variant *AIRE* alleles predicts progression to APECED. The *AIRE* gene maps on chromosome 21q22.3 with 14 exons; the encoded protein is 545 aminoacids in length and weighs 58 kDa [2]. More than 100 *AIRE* mutations have been identified to date (Human Gene Mutation Database-http://www.hgmd.cf.ac.uk; http://bioinf.uta.fi/AIRE-base/) within the exon/intronic sequence of the gene including single nucleotide substitutions or large deletions [23]. As highlighted above, some *AIRE* mutations that cause APECED are prevalent in certain populations [8, 10, 11, 20, 27–30]. Indeed, *AIRE* mutation c.769C > T (R257X) is prevalent in the Finnish population [19], c.415C > T (R139X) in the Sardinian [20] and c.254A > G (Y85C) in the Iranian Jew populations, suggesting a founder effect for these variants [12]. The R257X genotype was also found at high frequency in the Turkish population [21] and is overall responsible for 75% of alleles in patients with APECED in Central and Eastern Europe [16]. In particular, regarding the Italian population, 3 hot spots of incidence were identified, the first in Sardinia, the second in Apulia and the third in Veneto. In Sardinia, the presence of a peculiar *AIRE* gene mutation was identified and defined as R139X [20]. W78R was the prevalent mutation in Apulia and R203X in Sicily [28]. In Northern Italian populations, R257X mutation was reported frequently and often in association with mutation 1094-1106del13 [10]. These 2 mutations have been described in European populations [31].

On general ground, a genotype-phenotype correlation was observed only in a few populations. As regard to Iranian Jews, the nonsense Y85C mutation, CMC and AAD had low incidence and keratopathy was not observed [20]. Furthermore, the presence of the R257X mutation in Finnish patients was correlated with CMC [12, 20]. Of note, the autosomic dominant G228W mutation was discovered in a patient from Tuscany affected by CH and autoimmune thyroiditis with the presence of circulating 21OH antibodies [13].

In the Serbian APECED series of the present investigation, all genetically confirmed APECED patients (12 out of 13) harbored the homozygous R257X (p.Arg257X, c.769C > T) mutation in 10 out of 12 patients. Two patients presented the R257X mutation in heterozygosity with the c.462A > T (p.Glu298Lys) or the c.462A > T (p. (=)) mutation. These data suggest the presence of a founder effect for the R257X mutation in the Serbian population. No genotype/phenotype correlation was observed although all patients were affected by adrenal insufficiency and 10 out of 13 patients presented CMC. A high incidence of pernicious anemia among non-endocrine manifestations was also evidenced (Fig. 3).

## Conclusions

APECED is a rare autosomal recessive syndrome with biallelic mutations of *AIRE*. The different manifestations of disease vary significantly among patients with different ethnic backgrounds. The case report of the two siblings with APECED of the present investigation and the subsequent analysis conducted in the Serbian population highlight that the R257X genotype has a founder effect. The genotype was correlated with the occurrence of adrenal insufficiency in all patients. A high incidence of non-endocrine autoimmune manifestations was detected in the investigated cohort.

These data suggest a diagnostic workup that includes genetic screening for appropriate classification of APECED patients throughout different populations.

## Data Availability

Not applicable.

## Abbreviations

Abs: Autoantibodies
AAD: Autoimmune Addison’s disease
AADC: Aromatic l-amino acid decarboxylase
*AIRE*: AutoImmune Regulator
AITD: Autoimmune thyroid disease
APECED: Autoimmune polyendocrinopathy-candidiasis-ectodermal-dystrophy
APS-1: Autoimmune polyglandular syndrome Type 1
CH: Chronic hypoparathyroidism
CMC: Chronic mucocutaneous candidiasis
DNA: Deoxyribonucleic acid
HD: Histidine hydroxylase
L-T4: Levo-thyroxine
n: Number
POF: Premature ovarian failure
rev: Reviewed
OF: Ovarian failure
TPHAbs: Antibodis against tryptophan hydroxylase
TPO: Anti-thyroid peroxidase
yr: Years
